# Analysis of the mylohyoid nerve in elderly Japanese cadavers for dental implant surgery

**DOI:** 10.1002/cre2.341

**Published:** 2020-11-23

**Authors:** Shintaro Koga, Iwao Sato, Zhong‐Lian Li, Hidenobu Miyaso, Shinichi Kawata, Masahiro Itoh

**Affiliations:** ^1^ Department of Anatomy Tokyo Medical University Tokyo Japan

**Keywords:** communication, mandible, mylohyoid muscle, mylohyoid nerve, pain

## Abstract

**Objectives:**

Injury to the mandibular nerve (MN) branches may cause pain and irregular occlusal movement during mastication after mandibular dental treatments. Growing evidence indicates that the calcitonin gene‐related peptide (CGRP) plays a key role in the development of peripheral sensitization and the associated enhanced pain, suggesting it may be a sign to ensure a safe and reliable dental implant treatment. Our focus was on the distribution of the MN branches and their communication with the lingual nerve (LN), the localized expression of CGRP, and the identification of a pain area related to the mylohyoid muscle (MM) fascia in the mandibular floor.

**Material and Methods:**

In this study, MM samples from 440 sides of 303 human cadavers aged 61–103 years were examined microscopically and immunohistochemically. These data were further evaluated by the use of principal component analysis.

**Results:**

A complex but weak attachment site was identified for the fascia of the MM. CGRP expression was mainly located in small vessels and was scattered throughout the whole fascia of the MM. Communication between the MN and LN was found in 62.5% (275/440) of the samples. The results from the principal component analysis showed that the positive contributions were from the descending branch in the premolar region (correlation coefficient value *R* = 0.665), the ascending branch in the molar region (*R* = 0.709) and the intermediate branch of the digastric branch (*R* = 0.720) in component 1. In the fascia off the MM, strongly labeled CGRP‐positive cells were also found around the blood vessels and the nerve.

**Conclusions:**

The findings reported in this study indicate that there is a risk of damage when pulling the fascia off the MM at the border of the molar and premolar regions during dental implant surgery.

## 1 INTRODUCTION

The mandibular nerve is mainly composed of two roots that send branches to the mylohyoid muscle (MM) and the anterior belly of the digastric muscle. The posterior trunk of the MN gives rise to the main sensory nerves, that is, the auriculotemporal, lingual, and inferior alveolar nerves and motor branches to the MM and the anterior belly of the digastric muscle (Brennan, Webb, Kemidi, Spratt, & Standring, 2008). Communication between the MN and LN was also found at some frequency (46.3% Kameda & Uber den, 1952; Sato, Sunohara, Ueno, & Yoshida, 2004, 66%). In dental implant or oral surgery, the inner surface of the mandible has become an important area for lingual flap dental treatment (Urban et al., 2018).

Although morphological observations of the mylohyoid lines on the inner surface were reported recently (Aoki, Nara, & Kageyama, 2011), the mylohyoid lines are becoming the landmarks for peeling off part of the tissue attachment along the mylohyoid lines. Despite taking careful precautions, many studies have reported various injuries to the MN or LN in the mandibular bone and surrounding soft tissue during dental implant surgery (Del Castillo‐Pardo de Vera, López‐Arcas Calleja, & Burgueño‐García, 2008; Ferneini, Gady, & Lieblich, 2009; Frenken, Zijderveld, van den Bergh, Huisman, & Cune, 2010; Hofschneider, Tepper, Gahleitner, & Ulm, 1999; Jo, Kim, & Oh, 2011; Kalpidis & Konstantinidis, 2005; Kalpidis & Setayesh, 2004; Laboda, 1990; Mordenfeld, Andersson, & Bergström, 1997; Niamtu, 2001; Pigadas, Simoes, & Tuffin, 2009; ten Bruggenkate, Krekeler, Kraaijenhagen, Foitzik, & Oosterbeek, 1993; Woo, Al‐Bustani, & Ueeck, 2006). One of the main reasons for this may be that the detailed morphological characteristics of the branches from the MN have not been reported in human beings. Injuries to the MN branches may lead to pain or irregular movement of mastication after mandibular implant treatments and other dental operations (Alhassani & AlGhamdi, 2010; von Arx, Hafliger, & Chappuis, 2005).

A case report showed that the mylohyoid line somewhat deviates from that position, which makes precision peeling extremely difficult (a reference is needed here). Clinically, the submental triangle containing the MN and the submental artery is one of the important areas for dental implants or oral surgery using the lingual flap. Therefore, it is necessary to investigate the distribution of branches of the MN and the range of origin of the MM within the fascia.

The pain due to injury of the mandibular nerve branches may be related to local expression of the calcitonin gene‐related peptide (CGRP). In general, the blood flow in submental blood vessels is controlled by the sympathetic and parasympathetic nerves. In particular, the dominant effect of the sympathetic nerve on blood flow decreases at this site. There is a large amount of evidence showing that CGRP mainly controls the smooth muscle in the small branches of blood vessels, such as the facial, superficial temporal and maxillary arteries. Throughout these branches, CGRP restricts the blood supply to the submandibular triangle area. CGRP is also a neurotransmitter that helps transmit signals related to pain from the muscle's vessels (Ambalavanar et al., 2006; Azuma, Miwa, & Sato, 2016; Onuoha & Alpar, 1999; Sakuma et al., 2016). There are several mechanisms by which CGRP induces vascular relaxation (Bell & McDermott, 1996; Brain & Cambridge, 1996; Marshall, 1992). The distribution of CGRP affects pain and hearing through its actions on the human tensor tympani muscle (Yamazaki & Sato, 2014). Therefore, it is necessary to examine the localization of CGRP as a neurotransmitter at the site of pain in the submandibular triangle.

Our focus was on the distribution of the MN branches and their communication with the lingual branches at the microscopic level. We also observed the expression of CGRP at the inner and outer surfaces of the fascia using immunohistochemical methods, which might be related to pain during the reflection of the MM fascia in the submandibular triangle. All data, including the sex and the left–right differences, were further evaluated using principal component analysis (PCA). These results may provide useful information for safer oral clinical treatments.

## 2 METHODS

### 2.1 Ethics approval and informed consent

This study was performed in line with the principles of the Declaration of Helsinki (as revised in 2013). The cadavers used in the present study were donated to Tokyo Medical University and Nippon Dental University, Tokyo, Japan, based on the Act on Body Donation for Medical and Dental Education. All of the donors willingly signed a form consenting to the body donation for education and research, and all of the donors could revoke the intended donation at any time without any disadvantages. The collection and use of the mucosa data of all cadavers was approved by the donors and their families, and then carried out in accordance with the Law Concerning Cadaver Dissection and Preservation enacted in Japan in 1949. The study was approved by the Ethics Review Committee of Nippon Dental University (July 21, 2018; no. NDU‐T2015‐20) and Tokyo Medical University, Institutional Review Board (November 18, 2019; TMU, no. T2019‐0129).

### 2.2 Subjects and methods

In this study, mylohyoid and digastric muscles from 440 sides of 303 human cadavers aged 61–103 years (mean, 83.4 years) (178 male cadavers, 79–93 years old, mean 78.8 ± SD 8.29; 125 female cadavers, 61–103 years old, mean 85.6 ± 8.77) were examined. The cadavers were injected with 10% formalin with return perfusion through the femoral artery. The MM was dissected using a scalpel and tweezers under a microscope. We used 440 sides for the classification of the communication between the MN and LN. Moreover, we also used 70 sides to define the MN branch division points in detail. Some MMs with fascia (*n* = 8) were removed from the mandible at the microscopic level. These specimens were examined microscopically following analysis of the immunohistochemical stained sections.

### 2.3 Paraffin‐embedded sections of CGRP

Tissue sections of the outer surface and inner surface regions of the MM (cross‐section of the origin side of the middle zone) were deparaffinized with xylene and rehydrated through a series of ethanol solutions in PBS. Endogenous peroxidase was blocked with 0.3% H_2_O_2_ in methanol for 30 min, followed by incubation with Protein Block (Genostaff Co., Ltd. Japan) using an avidin/biotin blocking kit (Vector SP‐2001) (*n* = 8). These sections were then incubated with rabbit polyclonal antibodies against CGRP (10 μg/mL; ENZO BML‐CA1134) or normal rabbit IgG as a negative control for CGRP at 4°C overnight. After washing with TBST and TBS, they were incubated with biotin‐conjugated goat anti‐rabbit IgG (Dako E0432) at room temperature for 30 min. Then, the sections were incubated with peroxidase‐conjugated streptavidin (Nichirei, Tokyo, Japan) at room temperature for 5 min. Peroxidase activity was visualized using diaminobenzidine. The sections were counterstained with Mayer's hematoxylin (MUTO Pure Chemicals Co., Ltd. Japan), dehydrated, and then mounted with Malinol (MUTO Pure Chemicals Co., Ltd.). Images were acquired using a microscope (Leica DM 2500 16; Leica Microsystems) and Leica Application Suite software (Leica Microsystems).

### 2.4 Statistical methods

The data were assessed using a Pearson's chi‐square test to determine the validity of the distribution. The level of significance (*p* < .05) was calculated using Pearson's chi‐square tests. Principal component analysis (PCA) and cluster analysis were performed for the human MNs comprised of 13 elements, including age, sex, the difference between right and left, and the ratio of the MNs. These effects were assessed using one‐way analysis of variance (ANOVA) followed by Bonferroni's post hoc test with one categorical independent variable and one continuous variable. These results are reported as the mean ± *SD*. The statistical analyses were performed using IBM SPSS Statistics Base, version 22.

## 3 RESULTS

### 3.1 Attachment of the MM and mylohyoid line

**FIGURE 1:**
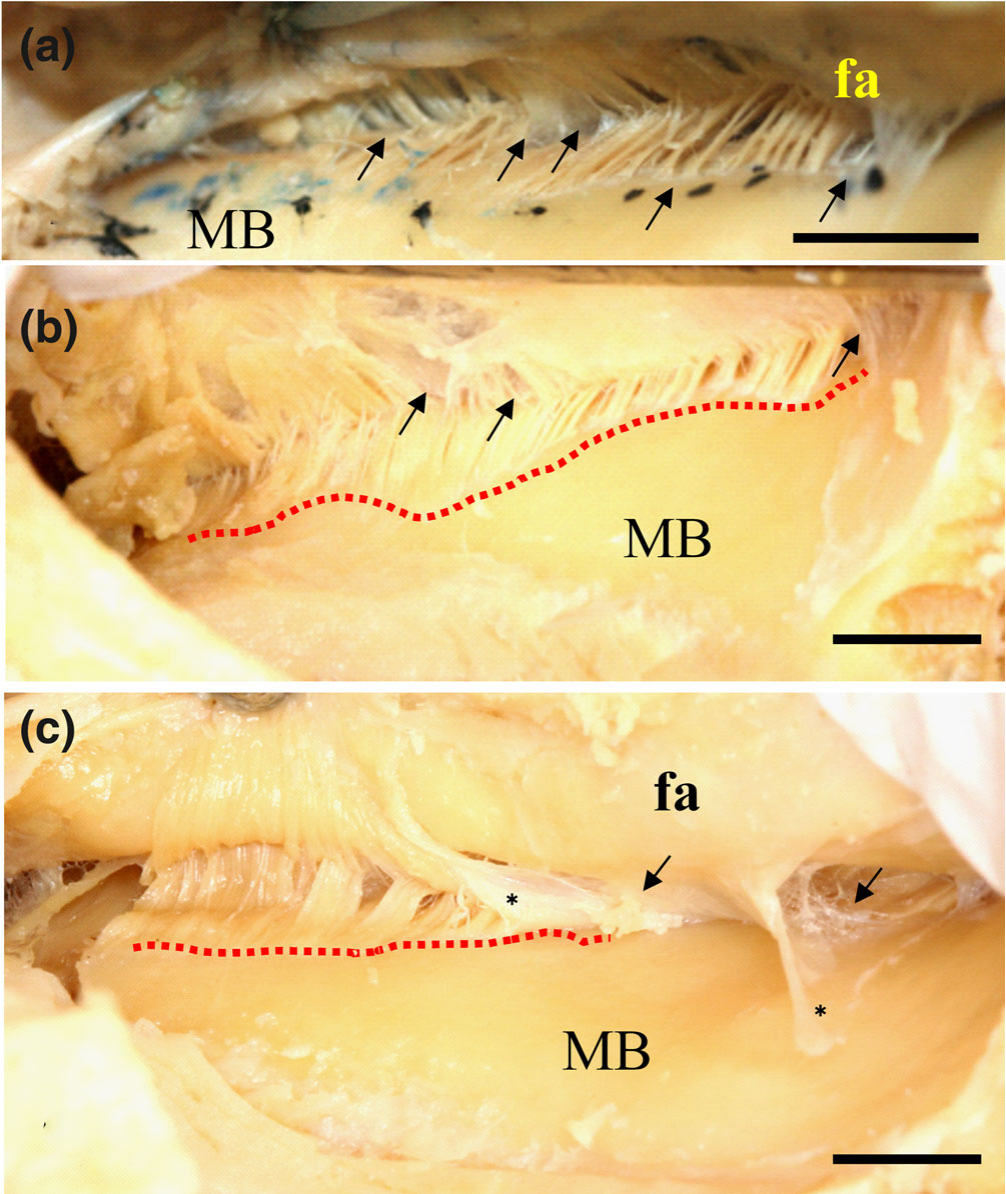
View of the fascia and some of the mylohyoid muscle (MM) attachments on the inner surface of the human mandibular body. The origin of the muscle fibers is shown by peeling the fascia (fa) off the MM from the mylohyoid line on the inner surface of the mandibular body (MB) (red dotted lines) (b,c). The site (red dotted lines) between the muscle fiber and thin serous membrane (*) is weakly attached at the mylohyoid line (arrows). (a) Attachment site of the MM; (b) Long site of attachment for the MM; (c) Short site of attachment for the MM; Ba = 200 mm; (a,b) dentulous samples; (c) edentulous samples. Bar = 1 cm

**FIGURE 2:**
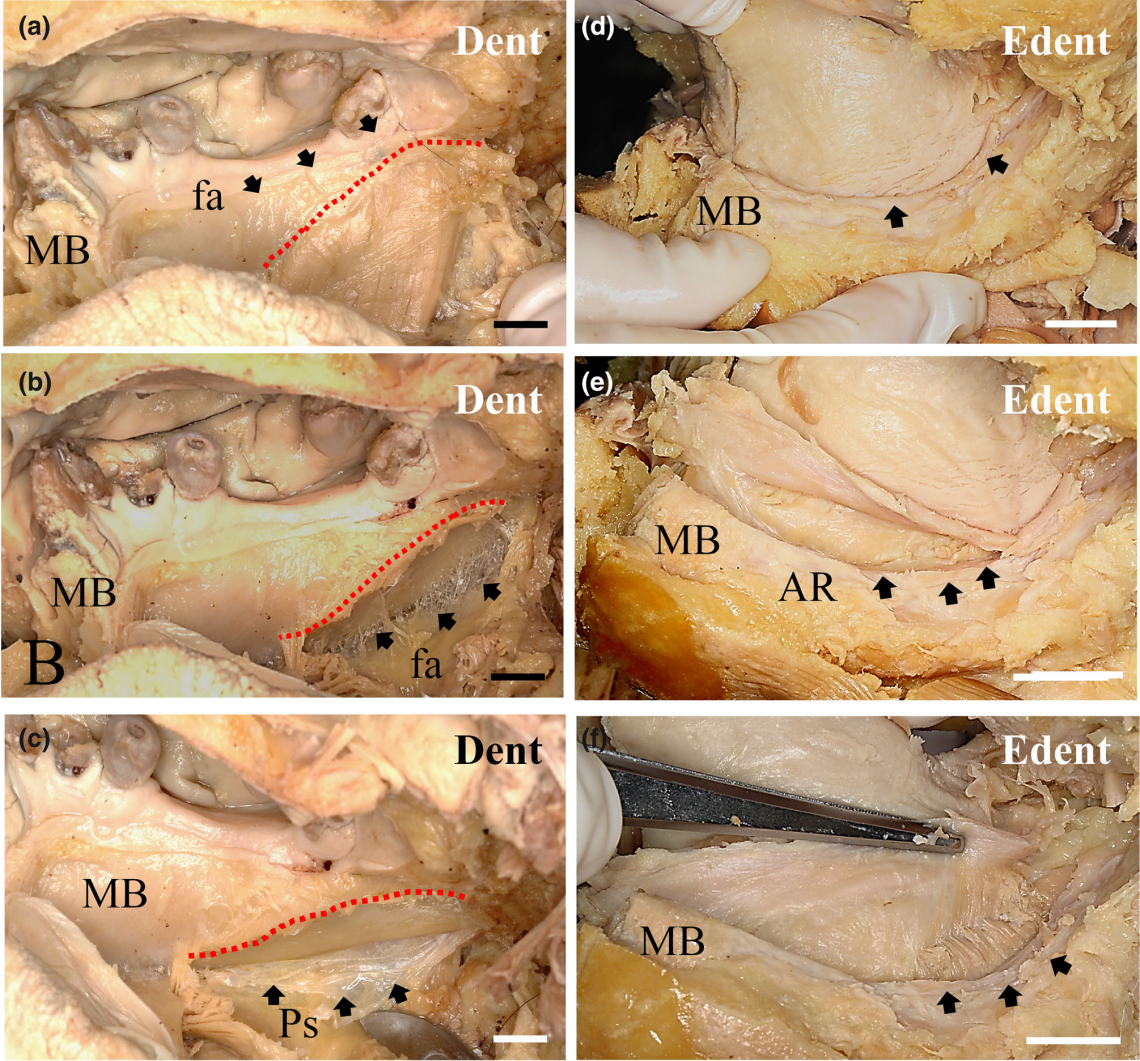
The attachment of the fascia covering the mylohyoid muscle (MM) in dentulous (Dent) and edentulous (Edn) samples. On the lingual side at the base of the mandible, the fascia of the MM appears as a wide and very thin sheet that covers the MM. The border of the MM origin is clearly different from the origin of the muscle, since that looks like a border line angled obliquely downward from the posterior region to the anterior regions (a). After peeling off the fascia of the MM from its origin along the mylohyoid line on the inner surface in the mandibular body, very fine and thin fascia‐composed fibers were found on the inner side of the mandible (b). The site between the muscle fiber and the thin serous membrane has a weak attachment at the mylohyoid line (arrows). Then, the periosteum (Ps) of the mandible body was removed from the mylohyoid line, and the small fine fibers were located between the periosteum and MM (c). On the lingual side at the base of the mandible, the border between the MM and the periosteum of the mandible body was almost indistinguishable at the site of the mylohyoid line (d). After peeling off the fascia of the MM from its origin along the mylohyoid line on the inner surface of the mandibular body, the periosteum of the mandible body was collected at this site, which is clearly not the fascia of the MM (e). Then, the periosteum of the mandible body was removed from the mylohyoid line, and the periosteum with thick connective tissue was collected between the periosteum and the MM (f). Red dotted line, origin of the MM; Bar = 1 cm. AR, alveolar ridge; fa, Fascia; MB, mandible

In the dentulous samples, the MM origin was located along the mylohyoid line on the mandible, which curved widely on the inner MM surface (Figure 1). After peeling off the fascia (fa), the attachment of the MM was clearly found from the molar region to the anterior basal region of the inner surface of the mandible (Figure 2). The fa with numerous fine fibers was also observed between the periosteum and surface of the mandible (Figures 1 and 2). In the edentulous region, the MM origin was limited, and the mylohyoid line receded and became shorter from the infraorbital foramen to the posterior region of the inner surface of the mandible (Figure 2). The fa of the MM was thin, and the periosteum was also found in the attachment area of the mylohyoid line. The alveolar ridge was found in the anterior region of the mandible (Figure 2).

### 3.2 Fascia structure

The origin of muscle fibers was found by peeling off the MM fascia from the mylohyoid line of the inner surface of the mandibular body (Figure 1). The site between the muscle fiber and the thin serosa was weakly attached to the mylohyoid line (Figure 1). The fascia covering the MM was also thin and formed a weak attachment border of the MM at the mylohyoid line on the inner surface of the human mandibular body. The muscle attachments at the site of the mylohyoid line were either long or short. A short attachment site was mainly observed at the inner surface of the anterior region of the edentulous human mandible in contrast to that of a dentulous human mandible (Figure 1). In edentulous samples, the attachment site was mainly observed in the limited mylohyoid line, where the cortical bone was irregularly formed and showed a resorption site compared to the findings in dentulous samples (Figures 1 and 2). In particular, the fascia was very thin and formed what appeared to be loose connective tissues in our observed samples on the inner side of the mandible (Figure 2). However, the fascia on the outer surface of the MM became gradually thicker from the anterior region to the posterior regions of the mandible (Figures 1 and 2).

### 3.3 Branches of the MN division points

**FIGURE 3:**
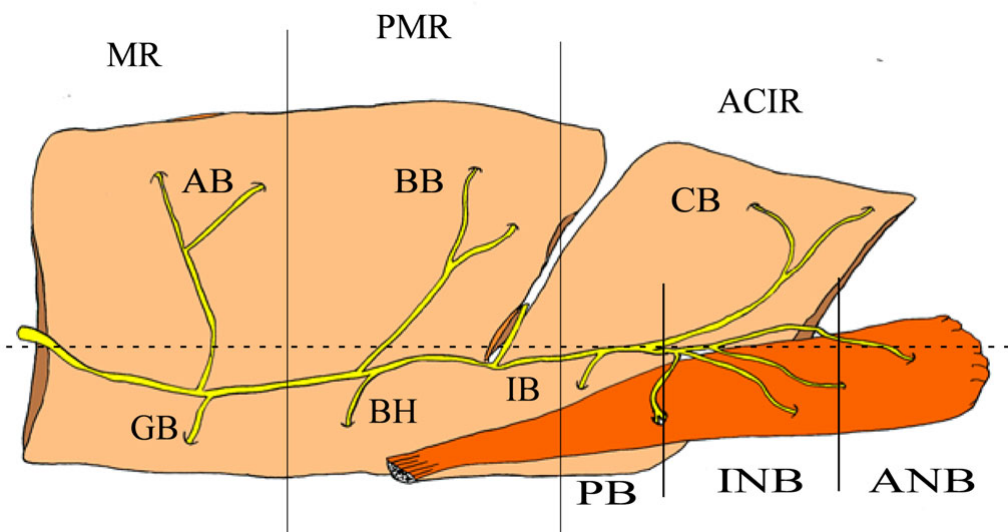
Schematic view of the mylohyoid muscle (MM) and the supply nerve branches on the outer surface of the MM. The incisor region (CIR), premolar region (PMR), and molar region (MR) were divided into three sections (lines): the branch of the mandibular nerve (MN) division points, such as the ascending (AB) and descending branch (GB) of the molar region; the ascending (BB) and descending branch (BH) of the premolar region; and the ascending (CB) and descending branch (IB) of the anterior canine and incisor regions (dotted line). We identified the digastric branch division points, such as the anterior branch (ANB), intermediate branch (INB), and posterior branch (PB) (lines)

We identified the branches of the MN division points, such as the ascending (AB) and descending branch (GB) of the molar region, the ascending (BB) and descending branch (BH) of the premolar region, and the ascending (CB) and descending branch (IB) of the anterior canine and incisor regions. The branches of the digastric branch division points (Figure 3), such as the anterior branch (ANB), intermediate branch (INB) and posterior branch (PB), were also observed in the incisor region (CIR), premolar region (PMR) and molar region (MR). Especially, we delineated the ascending branch of the molar region (AB, *n* = 42/318, 13.2%), the ascending branch of the premolar region (BB, *n* = 77, 24.2%), and the anterior canine and incisor regions (CB, *n* = 82, 25.8%) in the collected samples. The descending branch was defined in the molar region (GB, *n* = 14, 4.4%), the premolar region (BH, *n* = 20, 6.3%), and the anterior canine and incisor regions (IB; *n* = 13, 4.1%). Furthermore, the digastric branch division points, such as the anterior branch (ANB, *n* = 19, 6.0%), intermediate branch (INB, *n* = 39, 12.3%) and posterior branch (PB, *n* = 12, 3.8%) were identified (Figure 3).

### 3.4 Submental nerve supply and blood vessels

**FIGURE 4:**
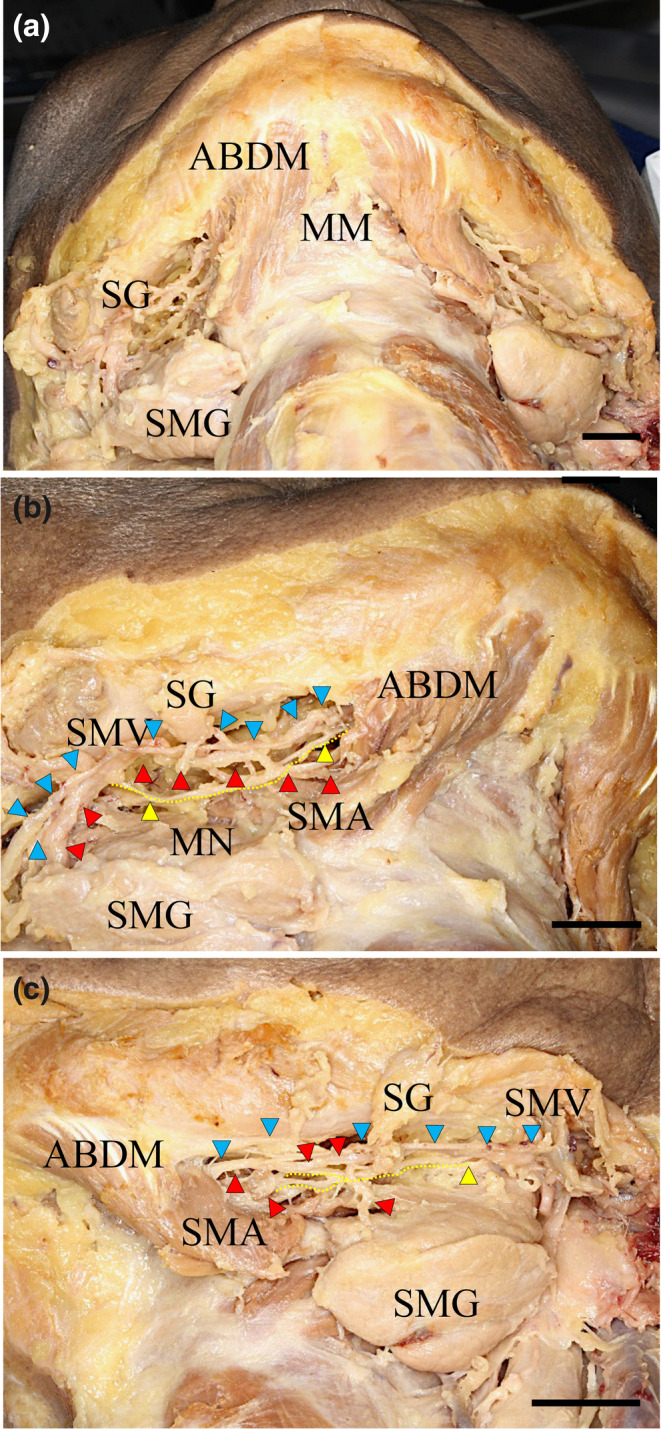
Microscopic features' view on the outer side of the mandibular floor with the submental nerve and artery and vein. (a) The submental nerve supplies the MM and runs along the submental artery and vein between the MM and the submandibular gland. (b) Right side and (c) left side, small fine MNs projected to the lateral surface of the MM. The submental nerves (yellow arrowheads, yellow dotted lines) ran along the submental artery (red arrowheads) and vein (blue arrowheads) between the MM and the submandibular gland. Some fine MNs run through a large slit between the muscle bundles of the MM and are connected to a branch of the LN. These fine MNs then projected to the lateral surface of the tongue. ABDM, anterior belly digastric muscle; MM, mylohyoid muscle; MN, mylohyoid nerve; SG, submandibular lymphatic ganglion; SMA, submental artery; SMG, submandibular gland; SMV, submental vein. Bar = 1 cm

**FIGURE 5:**
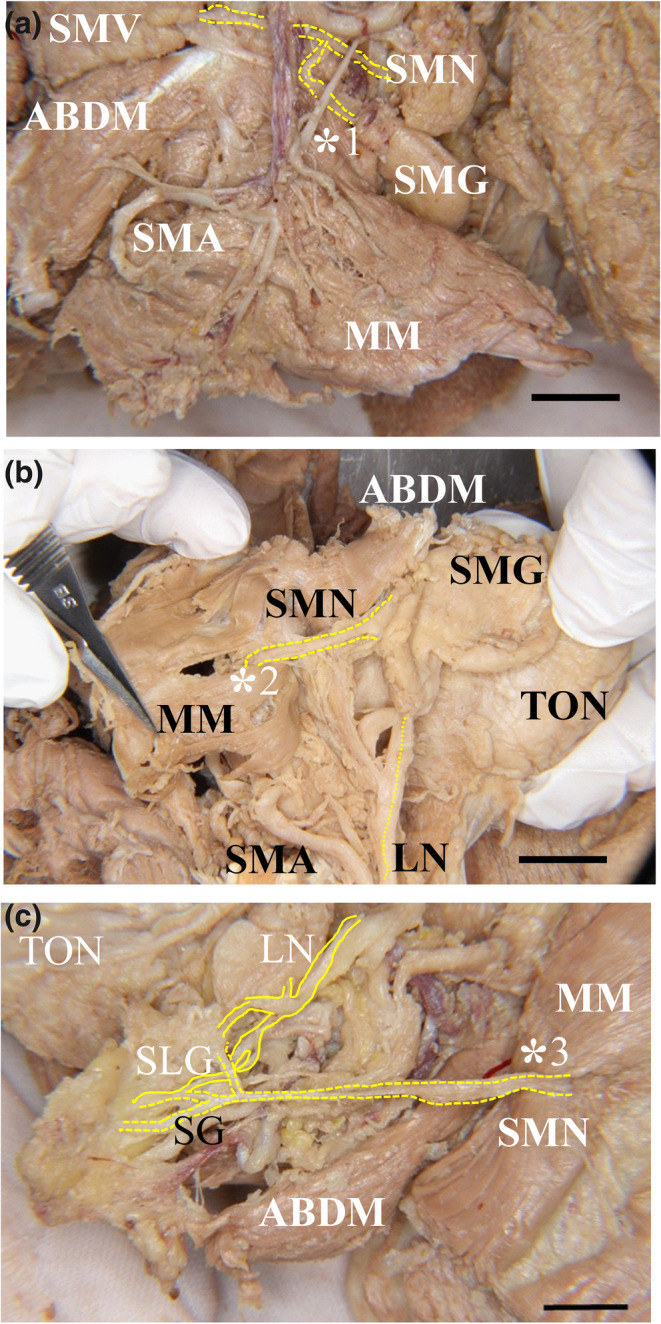
The communication between the MM and LN. The mandibular nerve (MN) is located at the most medial side of the submental vein (SMV) and artery (SMA) in the submandibular triangle. There were few MN branches except for the anterior region of the MM in this case (yellow dotted lines, *1–3). (a) Lateral view of the MM and anterior belly of the digastric muscle, (b) The inner side of the MM of (a) forming a communication pathway between the MM and LN (*2), (c) In the inner side of the MM, forming a clear communication pathway between the SMN and LN (*3). ABDM, anterior belly of the digastric anterior muscle; FA, facial artery; LN, lingual nerve; MM, mylohyoid muscle; SLG, sublingual gland; SMG, submandibular gland; SMN, submental nerve; TON, tongue. Bar = 1 cm

The submental nerve (SMN) descended to the posterior region in the MM. Many of the MM branches also continued to the submandibular gland in the inner surface of the MM, and a few branches extended from the posterior to the anterior base of the mandible with branches extending to the surface of the basal region of the MM. Therefore, we categorized the various directions of the MN branches into two portions: the anterior region (incisor regions, one third of the MM) and the posterior region (premolar and molar regions, two thirds of the MM). We also mainly found the ascending branch in the molar region, the ascending branch in the premolar region, and the ascending and descending branches (Figures 4 and 5) of the anterior canine and incisor regions. Some developed LNs traversed the anterior superior plane with respect to the tongue. On the other hand, the main trunk of the submental vein and artery were located in their basal regions near the hyoid bone. There were various branches of the submental artery in the posterior and the medial and anterior regions of the MM. These branches were divided from the main trunk at the hyoid bone body. The developed branches were mainly found in the anterior region at the surface of the MM (Figure 4). The medial branch of the submental artery ascended to the anterior superior region of the tongue after sending some fine branches to the lateral surface of the styloglossus muscle (Figure 5).

**FIGURE 6:**
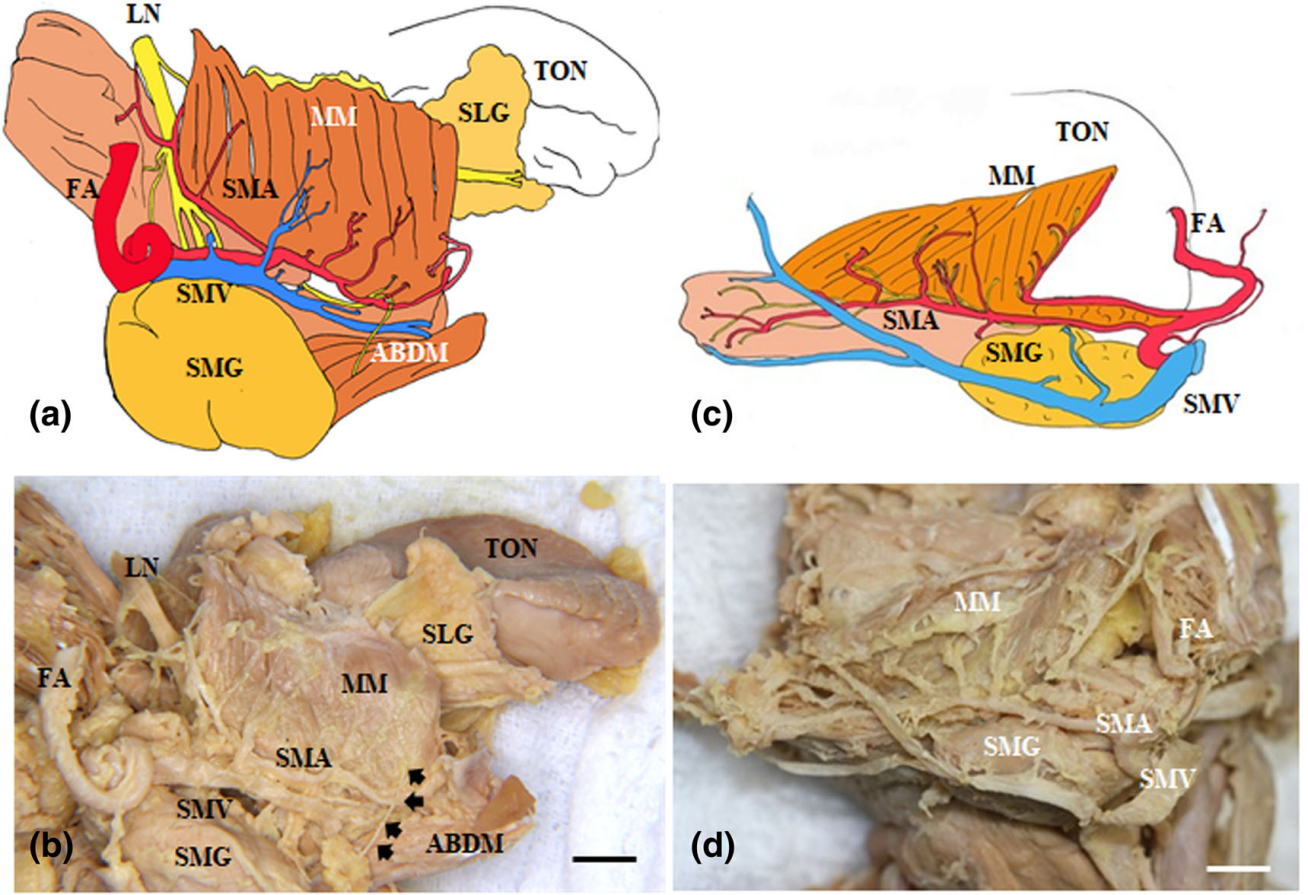
The submental nerve and submental vessels showing different innervations between the right and left sides in the same sample. The mandibular nerve (MN) is located at the most medial side of the submental vein (SMV) and artery (SMA) in the submandibular triangle. The MN branch projects to the MM. In the right and left views of the lateral side of the mandible, on the right side of the mandible (a,b), numerous branches of the MN (arrows) run along and innervate the MM in contrast to that of the left side of the MM (c,d). (a), Schematic of (b); (b) Lateral view of the right side of the MM and anterior belly of the digastric muscle. (c) Schematic of (d); (d) Lateral view of the left side of the MM and anterior belly of the digastric muscle. ABDM, anterior belly of the digastric anterior muscle; FA, facial artery; LN, lingual nerve; MM, mylohyoid muscle; SLG, sublingual gland; SMG, submandibular gland; TON, tongue. Bar = 1 cm

**FIGURE 7:**
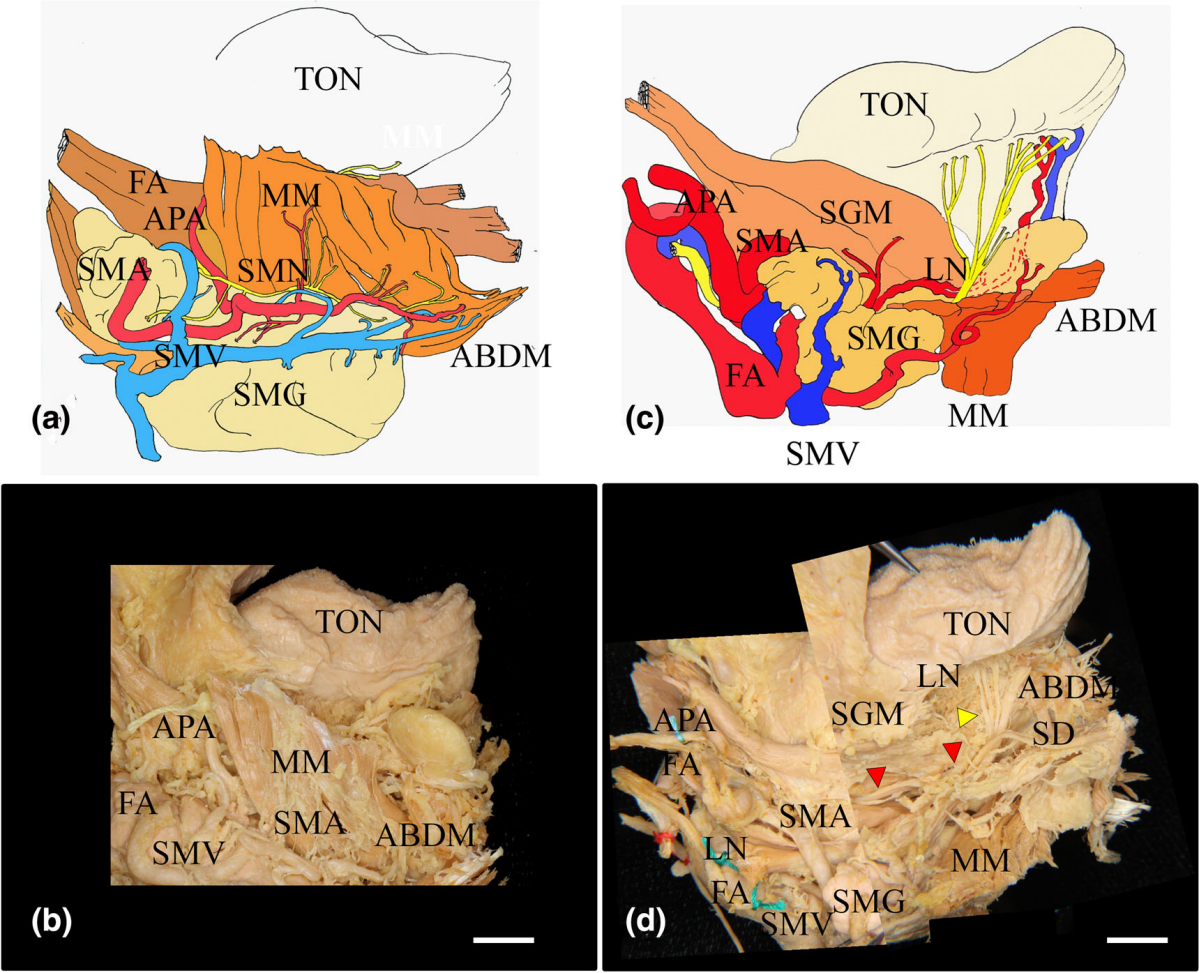
Microscopic features in the nonsublingual artery with a lateral view of the submental nerve, artery, and vein at the base of the mandible. In the lateral view of the basal region of the mandible, the submental nerve and artery and vein were located between the MM and anterior belly of the digastric muscle and the submandibular gland. The LN projected to the submandibular gland at the lateral surface of the MM. The main trunk of the submental vein and artery were clearly located in their basal regions near the hyoid bone (a,b). The MN was found in the upper region of the submandibular gland and extended many fine branches (arrowheads) to the MM. Various branches of the submental artery were mainly found in the posterior, medial, and anterior regions of the MM (a,b). On the medial side of the MM, the SMA branch (red arrowhead) extended to the lateral tongue through the MM. The LN (yellow arrowhead) was divided into branches in the lateral anterior region of the tongue (c,d). ABDM, anterior belly of the digastric muscle; APA, ascending pharyngeal artery; FA, facial artery; MM, mylohyoid muscle; MN, mylohyoid nerve; SD, sublingual duct; SG, submandibular lymphatic ganglion; SMA, submental artery; SMG, submandibular gland; SMV, submental vein. Bar = 1 cm

The MN was located at the basal region of the MM. Many small branches projected to the MM border area between the molar and premolar regions of the MM. Some of the SMN branches were ascending toward the surface of the anterior region of the MM, and a few small branches entered the MM and the anterior belly digastric muscle (Figure 6). One developed branch of the SMN ran through the middle region of the premolar region of the MM. After inverting the MM at the medial surface of the MM, a communication between the SMN and LN was found in the premolar region of the MM. The main LN clearly sent some branches to the submental gland (Figures 5 and 7). On the other hand, the main trunk of the submental artery was accompanied by the SMN to the basal region of the MM near the hyoid bone. In contrast, the main trunk of the submental vein was accompanied by the SMA at the surface of the submental gland. After inverting the MM to view the medial surface of the MM, we observed that the supplied submental artery extended to the lateral surface of the styloglossus muscle (Figure 6). The trunk of the innermost side of the submental vein was located beneath the innermost side of the submental artery.

### 3.5 Communication between the MN and LN

We identified the site of communications between the MN and LN. The communications between the MN and LN were mainly found in the premolar region (P2) of the human mandible (Figures 5 and 7). The ratio of the communication between the MN and LN (Com) was 62.5% (275/440). Negative correlations were found between the Com and BB in the MN (*r* > 0.283, *p* < .05).

### 3.6 The correlation coefficient measurement data, cluster analysis and PCA were performed for human MM in different groups for 11 variables including aging, sex, appearances, and locations of the MN supply

**FIGURE 8:**
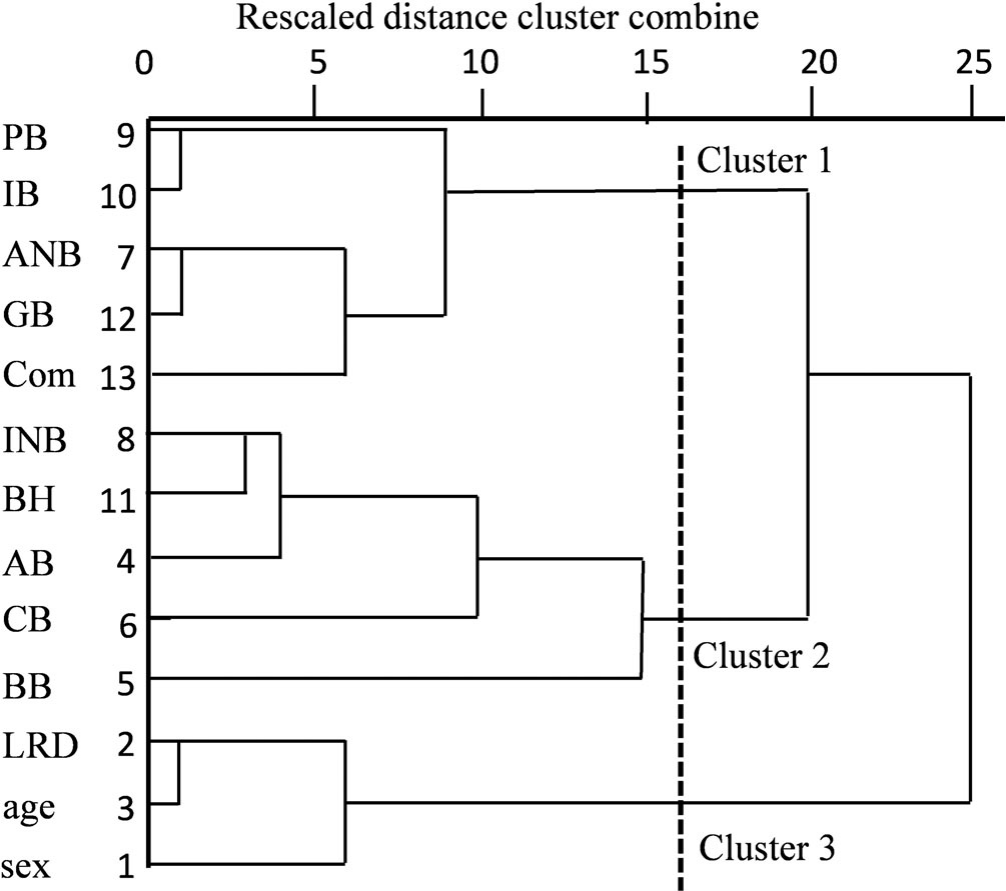
The dendrogram of variable factors obtained in all individual samples from the measured data, age, and sex by cluster analysis. The quality of the alignment of the three trees can be measured in this study (clusters 1–3; dotted line). The cluster 1 group was comprised of five elements (PB, IB, AMB, GB, and Com). The cluster 2 group was also comprised of five elements (INB, BH, AB, CB, and BB). The cluster 3 group was composed of three elements (LRD, age, and sex). MN division points such as the ascending (AB) and descending branch (GB) of the molar region, ascending (BB) and descending branch (BH) of the premolar region, and ascending (CB) and descending branch (IB) of the anterior canine and incisor regions are shown. We identified the branches of the digastric branch division points such as the anterior branch (ANB), intermediate branch (INB), and posterior branch (PB) in the incisor region, premolar region, and molar region. The quality of the alignment of the three trees can be measured in this study. Com, communication between the mandibular nerve and lingual nerve; LRD, the difference between the right and left

**FIGURE 9:**
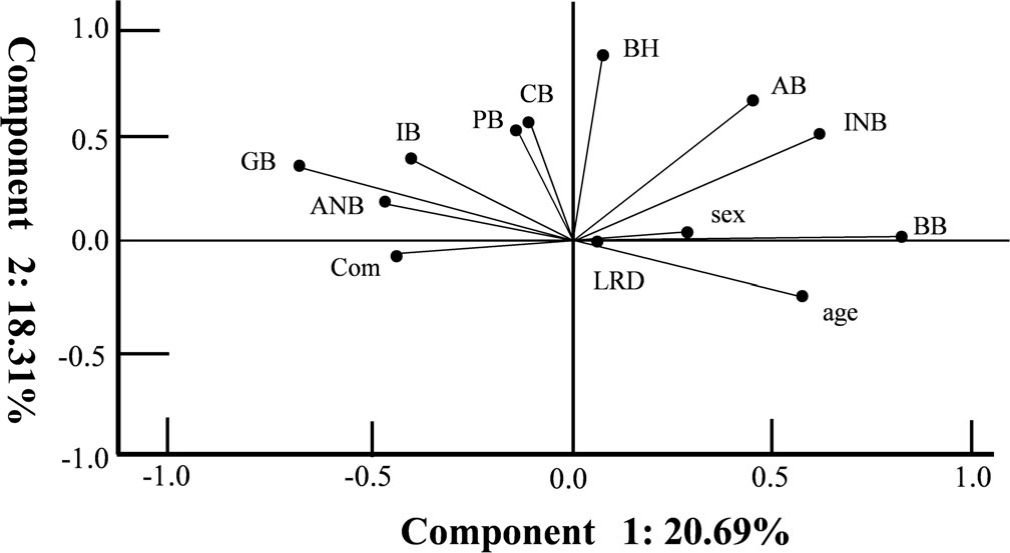
Variable factor map obtained in all individual samples from the measured data, age, and sex by principal component analysis (PCA). MN division points such as the ascending (AB) and descending branch (GB) of the molar region, ascending (BB) and descending branch (BH) of the premolar region, and ascending (CB) and descending branch (IB) of the anterior canine and incisor regions are shown. We identified the branches of the digastric branch division points such as the anterior branch (ANB), intermediate branch (INB), and posterior branch (PB) in the incisor region, premolar region and molar region. Com, communication between the mandibular nerve and lingual nerve; LRD, the difference between the right and left

There were correlation coefficients between 13 measured elements from the human MN based on the macroscopic data (Table 1). We performed clustering analysis using a hierarchical classification model with these two components, demonstrating that optimal grouping was obtained with three clusters (Figure 8). Cluster 1 included samples of PB, IB, ANB, GB and Com, whereas cluster 2 was exclusively comprised of INB, BH, AB, CB, and BB samples. Cluster 3 grouped samples of LRD, aging, and sex in the MM (Figure 8, 9).

**TABLE 1:** Correlation coefficients measurement data and PCA components were performed for human mylohyoid muscle

(A) Correlation coefficients measurement data													
	Sex	Age	AB	BB	LRD	CB	ANB	INB	PB	IB	BH	GB	Com
Sex	1												
Age	.356**	1											
AB	.00	−.032	1										
BB	−.007	.031	.282	1									
LRD	.077	.054	.00	−.028	1								
CB	.032	−.254	.402**	.148	.187	1							
ANB	−.156	−.093	−.383*	−.106	−.243	.416**	1						
INB	−.099	−.061	.490**	.282*	−.110	.166	.366*	1					
PB	.014	−.147	.340*	.144	.146	.418*	.173	.329	1				
IB	.093	−.144	.234	−.200	.155	.020	−.123	−.042	.468**	1			
BH	.065	−.073	.639**	.191	−.081	.547**	.311	.597**	.540**	.235	1		
GB	−.391*	−.396*	.271	−.200	−.091	.169	.626**	.194	.135	.345	.476**	1	
Com	−.077	−.218	−.323*	−.380*	−.119	−.039	−.144	−.154	.103	.154	−.050	.236	1

(B) PCA components was performed for human mylohyoid muscle													
	INB	AB	BB	BH	Com	GB	CB	Age	IB	PB	ANB	Sex	LRD
Component 1	**.720**	**.709**	**.665**	**.653**	**−.643**	−.269	.206	.352	−.050	−.202	−.167	.368	.134
Component 2	.294	.421	−.390	.578	.345	**.695**	**.538**	**−.532**	.387	.353	.482	−.260	−.134
Component 3	−.144	.003	−.181	.082	.300	−.061	−.351	−.085	**.696**	**.636**	**−606**	**.526**	.439

*Note:* Ascending (AB) and descending branch (GB) of molar region, ascending (BB) and descending branch (BH) of premolar region, ascending (CB) and descending branch (IB) of anterior canine and incisor regions, anterior branch (ANB), intermediate branch (INB), and posterior branch (PB), Com, communication between the mandibular nerve and lingual nerve (see Figures 4 and 9). *p < 0.05; **p < 0.01

The variables were plotted in a two‐dimensional space defined by two axes: component 1 (*x*‐axis) and component 2 (*y*‐axis). The two principal components significantly explained 39.0% (component 1, 20.69%; component 2, 18.31%) of the information in the data set of the human MN supply. In the MM, the most important variable explaining component 1 was BB. The first component illustrated the correlation between BB and INB, AB, and BH, and the negative correlation between BB and Com. Component 1 was explained mainly by the CB and PB, IB, GB, and ANB, and the negative correlation between aging and these groups. Figure 9 shows a display of the MM variables and components 1 and 2 on a two‐dimensional map (Table 1). We also observed positive contributions from BB (correlation coefficient 0.665), INB (0.720), BH (0.653), and AB (0.709), while negative contributions were observed for Com (−0.643) in component 1. In contrast, component 2 was defined by the expression of GB (0.696) and PB (0.636). Moreover, component 3 was defined by the expression of IB (0.597), PB (0.636), and sex (0.526), and negative contribution of ANB (−0.606) (Figure 9 and Table 1). The following correlations among the appearance ratio of the MN branches of the MM are shown in Table 1: We observed a fine positive correlation between BH and PB (*r* > 0.627, *p* < .01), CB (*r* > 0.624, *p* < .01), AB (*r* > 0.581, *p* < .01), and INB (*r* > 0.437, *p* < .01); a positive correlation between GB and ANB (*r* > 0.662, *p* < .01) and a positive correlation with BH (*r* > 0.568, *p* < .01); a negative correlation between GB and age (*r* > −0.476, *p* < .01); a correlation between ANB and CB (*r* > 0.428, *p* < .01) and PB and CB (*r* > 0.532, *p* < .01); a correlation between IB and PB (*r* > 0.501, *p* < .01); a positive correlation between CB and AB (*r* > 0.421, *p* < .01); and a negative correlation between CB and aging (*r* > −0.325, *p* < .01) (Table 1).

### 3.7 Immunohistochemical observations of the localization of CGRP in whole mount samples and tissue sections with MM fascia

**FIGURE 10:**
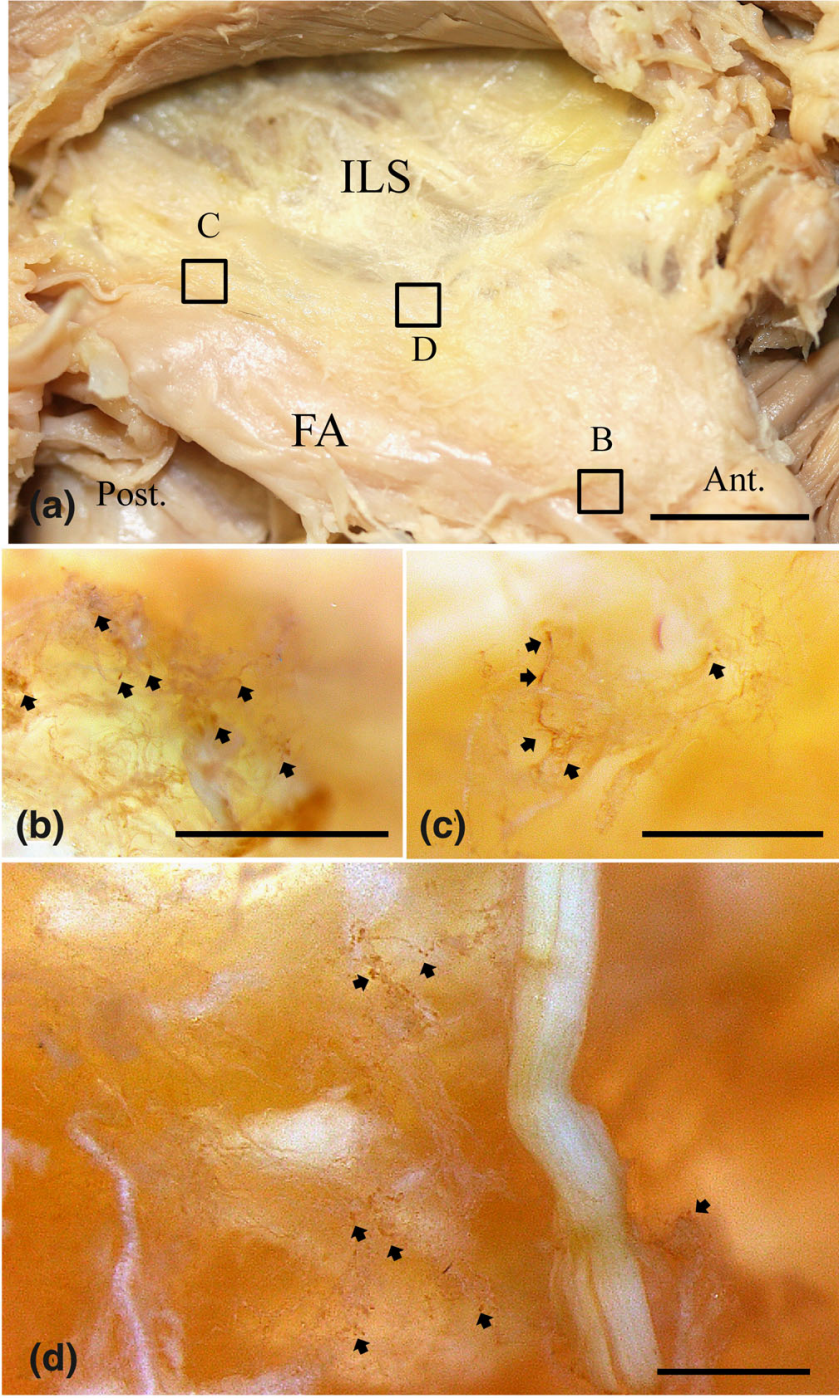
View of the reflection of the fascia of the mylohyoid muscle (MM) on the inner surface of the human mandibular body showing CGRP‐positive immunohistochemical staining in a whole mount sample. (a) The origin of the muscle fibers was seen by peeling off the fascia of the MM from the mylohyoid line of the inner surface of the mandibular body (MB). The sites (squares, b–d) are shown in the CGRP reaction areas in (a). The CGRP‐positive sites are found on some fine nerves of the fascia of the MM. The CGRP‐positive sites (arrows) were also mainly located in the large and small vessels (b–d). (b) In the posterior surface region of the mandibular body, small clusters of positive reactions (arrows) were concentrated in the middle zone of the fascia of the MM. (c) In the middle surface region of the mandibular body, small fine fibers that were positive for CGRP (arrows) were found on the fascia of the MM. (d) In the anterior surface region of the mandibular body, small fine fibers that were positive for CGRP (arrows) were found on the fascia of the MM. Ant, anterior region; ILS, inner lingual surface; Post, posterior region. Bar = 1 mm

**FIGURE 11:**
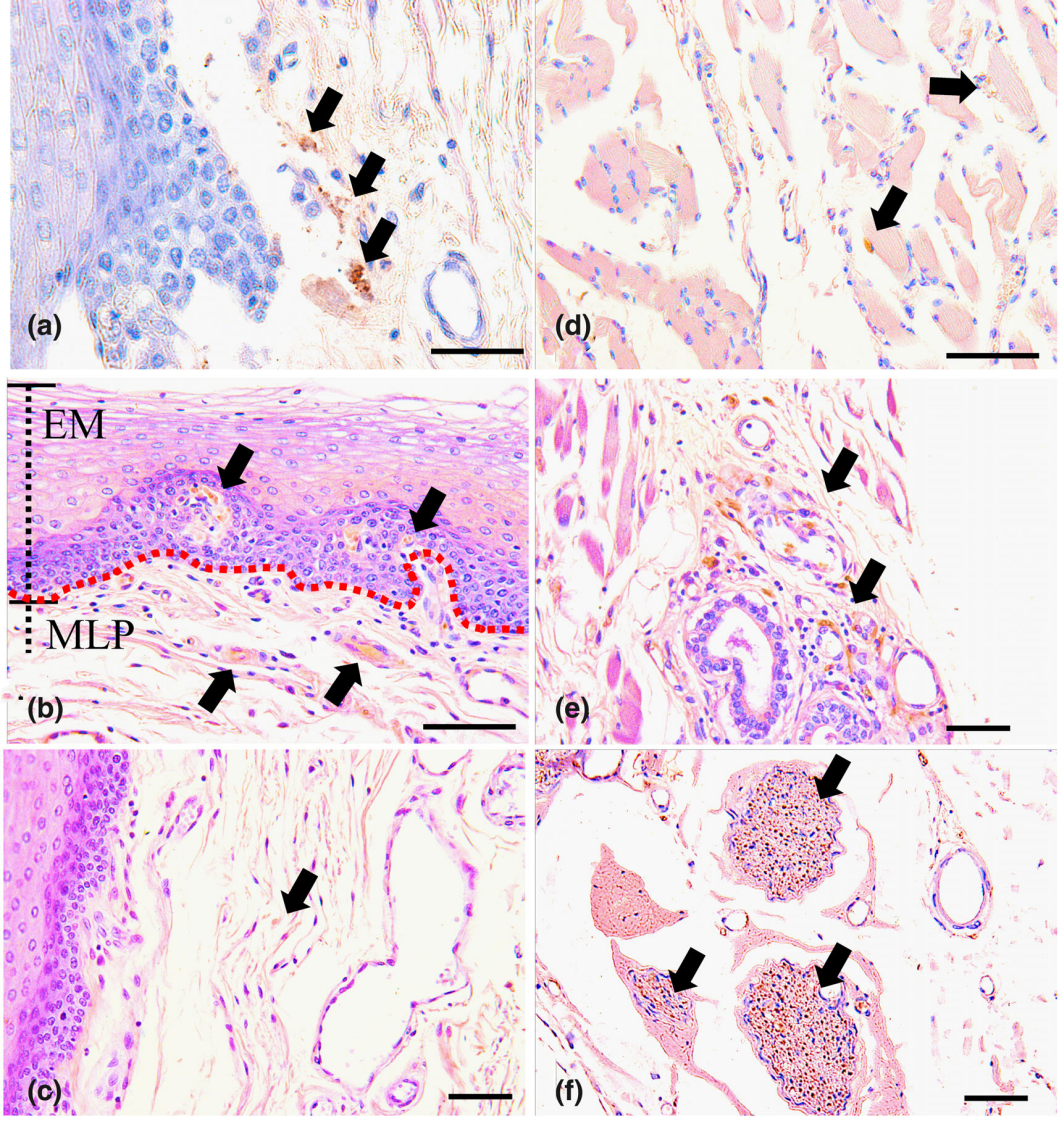
The localization of CGRP in immunohistochemically stained sections of the inferior lingual mucous membrane of the mylohyoid muscle (MM). Strongly labeled CGRP‐positive cells (arrows) were found in the mucosal lamina propria beneath the basal layer of the mucosal epithelium of the anterior and posterior regions of the mucosa of the MM (a,b). Fine positive fibers (arrows) were observed around the blood vessels in the anterior region of the mucosa of the MM (c). Small positive cells (arrows) were identified around mandibular glands and muscle fibers and in the fiber bundle of the nerves of the posterior region of the mucosa of the MM (d–f). (a) Anterior region of the mucosal epithelium, bar = 40 μm; (b) posterior region of the mucosal epithelium, the red dotted line indicates the border between the epithelial mucosa layer (EM) and the mucosal lamina propria layer (MLP), bar = 50 μm; (c) anterior region of the mucosal lamina propria, bar = 50 μm; (d) posterior region of the muscle fibers beneath the mucosal lamina propria, bar = 100 μm; (e) posterior region of the submandibular glands beneath the mucosal lamina propria, bar = 100 μm; (f) posterior region of the nerve bundles beneath the mucosal lamina propria, bar = 50 μm

In the fascia of the mylohyoid muscle (MM), the c scatter was sometimes located around the blood vessels. In the lateral region of the tongue, the CGRP reaction was mainly located at small cells around the vessels beneath the submucosa and lamina propria. Moreover, a fine positive reaction was found around the muscle fiber of the MM at whole mount analysis levels (Figure 10). In addition, we defined a few CGRP‐positive cells in the mucosal lamina propria beneath the basal layer of the mucosal epithelium in the anterior region of the base of the mandible in contrast to that of the posterior region. In the posterior region, some cells that were clearly positive for CGRP were mainly localized to the papillary zone of the mucosal epithelium, around blood vessels of the MM and the stained portion of the submandibular gland duct. Moreover, these strongly labeled CGRP‐positive cells were also detected in the nerve fiber or around the fiber bundle of the nerve by evaluating the tissue slice (Figure 11).

## 4 DISCUSSION

In the present study, detailed observations were carried out on the innervation and arrangement of the MN traversing in various directions. The number of nerve branches entering the MM was evidently more than that of the anterior belly of the digastric muscle. Communications between the MN and LN were mainly found between the premolar and molar region of the mandible, which has been shown to occur at a frequency ranging from 45 to 66% (46.3% Kameda & Uber den, 1952; 66% Sato et al., 2004, this study, 63%). It is interesting to point out that the communication occurs between the LN and MN, both of which originate from the mandibular nerve. The tongue is supplied by the facial, glossopharyngeal, hypoglossal and vagus nerves. This communication makes the innervation system of the tongue even more complicated, and such communication makes it more susceptible to injury during third molar extractions (Potu, D'Silva, Thejodhar, & Jattanna, 2010). It has been reported that during dental surgery, pain and dysesthesia resulting from injury to the LN would spread to the inner side of the tongue via the aforementioned system (Chossegros, Guyot, Cheynet, Belloni, & Blanc, 2002), which is consistent with our findings.

In general, the posterior region of the MM is in the vicinity of the molars, including the first and second molar teeth. It has been reported that the molar tooth location is the most important region for dental implant treatment (Huang et al., 2015; Lin et al., 2014). In the present study, the attachment site for the MM was clearly different between the dentulous and edentulous samples. In particular, structural differences in the MM fascia were found between the inner and outer side of the mandible. Despite the existence of many morphological risk factors during dental implant surgery (Lin et al., 2014; Moraschini & Porto Barboza, 2016; Nassar et al., 2014), our findings indicate that the difference in functional mastication should be a good guide to carefully observe the MM origin site on the mandible. Especially, when peeling off the fascia, the inner side of the MM fascia might be damaged easily due to its very fine and thin structure.

On the other hand, it is generally accepted that CGRP is a nerve pain marker in the smooth muscle of blood vessels (Ambalavanar et al., 2006; Azuma et al., 2016; Onuoha & Alpar, 1999; Sakuma et al., 2016) related to vascular relaxation (Bell & McDermott, 1996; Brain & Cambridge, 1996; Marshall, 1992). The location of CGRP indicates vasodilation or widening of the blood vessels related to the blood flow in human MM. CGRP is most frequently present among high‐threshold mechanosensitive (presumably nociceptive) afferent neurons, suggesting that CGRP is also expressed in many other types of primary afferent neurons (Hoheisel, Mense, & Scherotzke, 1994). It has been reported that most CGRP‐positive fibers are located in the outer layer of the fascia and the subcutaneous tissue around the MM (Tesarz, Hoheisel, Wiedenhöfer, & Mense, 2011). In the present study, the expression of CGRP was mainly detected around numerous vessels in the MM fascia and in the posterior region of the MM. The expression of CGRP at different locations may be a landmark that signifies a pain risk for peeling off the MM fascia and the periosteum of the mandible during oral surgery. On the lingual side of the scheduled implant site, a preoperative biopsy of the oral mucosa on the mylohyoid muscle could be performed to examine the positive activity level of CGRP, which might provide information about the prognosis and help guide the procedure. It has also been reported that CGRP originating from the trigeminal ganglion cells could amplify nociception and lead to migraines (Deen et al., 2017; Dussor et al., 2014). Therefore, rimegepant, which is a CGRP antagonist, and fremanezumab, which is a monoclonal antibody targeting CGRP, have been developed (Lipton et al., 2019; Scuteri et al., 2019). It would be interesting to determine if local administration of these reagents could suppress migraine vasodilatation and headaches.

In the cluster analysis, these elements were classified into three groups; cluster 1 comprised of PB, IB, ANB, GB, and Com; cluster 2 comprised of INB, BH, AB, CB and BB; and cluster 3 comprised of LRD, age and sex. In component 1, positive contributions were defined from INB, AB, BB, and BH, and in contrast, negative contributions were from Com. These elements are the most important nerve supplies to the middle region of the MM and digastric muscle. Moreover, these nerve branches extend to the origin of the digastric anterior muscle and the MM, which may be related to the pain factors after pulling the fascia off the MM. These elements also had a negative correlation with the Com, and the appearance of the Com is related to these elements in the reduced number of nerves on the BH, AB, INB, and BB. Component 2 was defined by the CB, GB, IB, and ANB, which had positive contributions in contrast to the negative contribution to age and LRD. A reduction in the number of nerves may also be related to the occlusion due to missing teeth associated with aging. Therefore, our PCA indicated the MN branches priority and importance and the correlations in the MN of the MM.

Moreover, one of the purposes of this study was to accurately identify the MN pathway using some anatomical landmarks in its vicinity. In general, severe trigeminal neuralgic pain mainly occurs, compared to the pain that occurs via any other nerve in the body (Benoliel & Eliav, 2008; DuPont, 2008). The distribution of the fine small vessel supplies is also important for muscle pain conception (Wasner et al., 2002). We found that the attachment site of the MM was placed along the mylohyoid line, along which the fascia curved widely on the inner surface of the MM at the mandible. After peeling off the fascia, the attachment site of the MM was clearly found from the molar region to the anterior basal region at the inner surface of the mandible. The fascia with numerous fine fibers was also found between the periosteum and the surface of the mandible. In the edentulous region, however, the attachment site of the MM was limited, and the mylohyoid line receded and became shorter from the location of the infraorbital foramen to the posterior region at the inner surface of the mandible. Although the fascia of the MM was not clear, the periosteum was also found in the attachment area of the mylohyoid line. The alveolar ridge was observed in the anterior region of the mandible. Our results indicate that even though the mylohyoid lines are becoming the landmarks when peeling part of the MM mylohyoid line during implant treatment, the origin of the MM is not consistent with this mylohyoid line. A case report showed that the MM somewhat deviates from that position, which makes the peeling extremely difficult. Therefore, the distribution of the MN branches and the range of origin of the MM should be considered important landmarks for mandibular dental treatments.

## 5 CONCLUSIONS

We investigated the MN branches supplying the MM and the anterior belly of the digastric muscle. In addition to the most frequently existing MN branches in the premolar and molar regions, we found communications between the MN and LN (62.5%, 275/440), which had a negative correlation with the appearance of BH, AB, INB, and BB by use of PCA. Our findings indicate that the expression of CGRP might be a marker that signifies a pain risk for pulling the fascia off the MM in the border of the molar and premolar region during dental implant surgery.

## CONFLICT OF INTEREST

The authors declare no conflicts of interest.

## AUTHOR CONTRIBUTIONS

Shintaro Koga, Iwao Sato, and Masahiro Itoh conceived the work. Shintaro Koga and Iwao Sato designed the work. Shintaro Koga, Iwao Sato, Zhong‐Lian Li, Shinichi Kawata, and Hidenobu Miyaso interpreted the data. Iwao Sato, Zhong‐Lian LI, Shinichi Kawata, and Hidenobu Miyaso acquired the data. Masahiro Itoh drafted the manuscript. All authors revised the work, gave final approval of the version to be published, and agreed to be accountable for all aspects of the work.
